# Identification of neuromedin U precursor-related peptide and its possible role in the regulation of prolactin release

**DOI:** 10.1038/s41598-017-10319-9

**Published:** 2017-09-05

**Authors:** Kenji Mori, Takanori Ida, Mami Fudetani, Miwa Mori, Hiroyuki Kaiya, Jun Hino, Keiko Nakahara, Noboru Murakami, Mikiya Miyazato, Kenji Kangawa

**Affiliations:** 1 Department of Biochemistry, National Cerebral and Cardiovascular Center Research Institute, Suita Osaka, 565-8565 Japan; 2 Division of Searching and Identification of Bioactive Peptides, Department of Bioactive Peptides, Frontier Science Research Center, University of Miyazaki, Miyazaki, Miyazaki 889-1692 Japan; 3 Division of Research & Inspection for Infectious Diseases, Center for Animal Disease Control, University of Miyazaki, Miyazaki, Miyazaki 889-1692 Japan; 4 Department of Veterinary Physiology, Faculty of Agriculture, University of Miyazaki, Miyazaki, Miyazaki 889-2192 Japan

## Abstract

The discovery of neuropeptides provides insights into the regulation of physiological processes. The precursor for the neuropeptide neuromedin U contains multiple consensus sequences for proteolytic processing, suggesting that this precursor might generate additional peptides. We performed immunoaffinity chromatography of rat brain extracts and consequently identified such a product, which we designated neuromedin U precursor-related peptide (NURP). In rat brain, NURP was present as two mature peptides of 33 and 36 residues. Radioimmunoassays revealed NURP immunoreactivity in the pituitary, small intestine, and brain of rats, with the most intense reactivity in the pituitary. Intracerebroventricular administration of NURP to both male and female rats robustly increased plasma concentrations of prolactin but not of other anterior pituitary hormones. In contrast, NURP failed to stimulate prolactin release from dispersed anterior pituitary cells. Pretreatment of rats with bromocriptine, a dopamine receptor agonist, blocked the prolactin-releasing activity of NURP. In rats pretreated with the antagonist sulpiride, intracerebroventricular administration of NURP did not increase plasma prolactin concentrations more than administration of saline. These data suggest that NURP induces prolactin release by acting indirectly on the pituitary; dopamine from the hypothalamus, which inhibits prolactin release, may be involved in this activity of NURP.

## Introduction

Neuropeptides and peptide hormones are major signalling molecules responsible for cell-to-cell communication and play pivotal roles in regulating a variety of physiological phenomena. Neuropeptides and peptide hormones are synthesized as inactive precursor proteins that undergo post-translational modification to generate the biologically active peptides^1^. An important step in this modification is limited endoproteolytic cleavage of the precursor, which mostly occurs at the C-terminal side of paired basic residues, such as Arg–Arg and Lys–Arg; these paired residues are the typical consensus sequences for cleavage catalysed by the subtilisin-like proprotein convertases^1^. Although many neuropeptides and peptide hormones have been discovered by purification from tissue extracts that display a biological activity of interest, several have been identified on the basis of common structural features, including consensus sequences for processing of the precursor^2–5^. For example, the Arg–Phe–amide peptide family and salusins were discovered by bioinformatic analysis of cDNA sequences^2, 3^. Moreover, proadrenomedullin N-terminal 20 peptide and neuronostatin were discovered because the amino acid sequences of their peptide precursors, preproadrenomedullin and preprosomatostatin, contain multiple cleavage sites composed of basic residues^4, 5^.

Neuromedin U (NMU), initially isolated from porcine spinal cord by our group, is a brain–gut neuropeptide that has potent activity for contracting uterine smooth muscle^6^. It activates NMU receptors types 1 (NMUR1) and 2 (NMUR2), which were previously identified as the orphan G protein–coupled receptors (GPCRs) FM-3/GPR66 and FM-4/TGR-1, respectively^7–10^. Although *NMU* mRNA is highly expressed in the pituitary and intestinal tract, with moderate expression in other tissues, including brain^8^, the predominant expression of *NMUR2* mRNA in the central nervous system has led to our understanding of the central function of NMU^9–11^. Intracerebroventricular (ICV) administration of NMU causes several biological responses, including suppression of feeding and increases in energy expenditure^7, 9, 12, 13^.

In another previous study, we identified the novel 36-residue neuropeptide neuromedin S (NMS), which activates both NMUR1 and NMUR2 in a cell-based assay in a manner similar to NMU^14^. *NMS* mRNA primarily is present in the brain, spleen, and testis. In rat brain, *NMS* mRNA is predominantly expressed in the suprachiasmatic nuclei, with low expression in other hypothalamic nuclei. Although ICV administration of NMS or NMU to rats induces the same responses, the activity of NMS is approximately ten times that of NMU in the brain^14–17^. NMU and NMS have an identical C-terminal amidated heptapeptide sequence that is essential for their agonist activities^6, 14^. However, NMS is not a splicing variant of NMU: the precursors for NMU and NMS are encoded by separate genes that map to human chromosomes 4q12 and 2q11.2, respectively^14^.

The domain structures of the precursors for NMU and NMS are remarkably similar and include four conserved consensus cleavage sites. NMU and NMS are flanked by the third and fourth cleavage sites in their respective precursors^14^. The amino acid sequences of these precursors exhibit some homology to each other between the first and second sites, and these regions show no homology to previously known peptides. Therefore, other novel neuropeptides may be produced from the NMU and NMS precursors by proteolytic cleavage at both the first and second sites, but biochemical evidence for such putative neuropeptides is unavailable currently.

In this study, to determine whether a novel neuropeptide is derived from the NMU precursor, we prepared an antiserum against the synthetic putative peptide and then purified two forms of the endogenous peptide from rat brain and small intestine by immunoaffinity chromatography followed by reversed-phase high-performance liquid chromatography (RP-HPLC). We named this peptide neuromedin U precursor-related peptide (NURP), and we were able to detect it immunochemically by radioimmunoassay (RIA). Because the *NURP/NMU* mRNA is highly expressed in the hypothalamo-pituitary region^9, 11^, we investigated the effect of NURP on the release of anterior pituitary hormones. ICV administration of NURP to rats specifically increased plasma prolactin concentrations, although NURP failed to stimulate the secretion of prolactin from dispersed anterior pituitary cells *in vitro*. In addition, ICV-administered NURP failed to alter plasma prolactin concentrations in rats pretreated with a dopamine receptor antagonist to disable the dopaminergic inhibition of prolactin release from lactotrophs. These data suggest that NURP stimulates prolactin release by acting indirectly on the lactotrophs of the pituitary and that dopamine from the hypothalamus may be important for this effect.

## Results

### Structure of NURP

**Figure 1:**
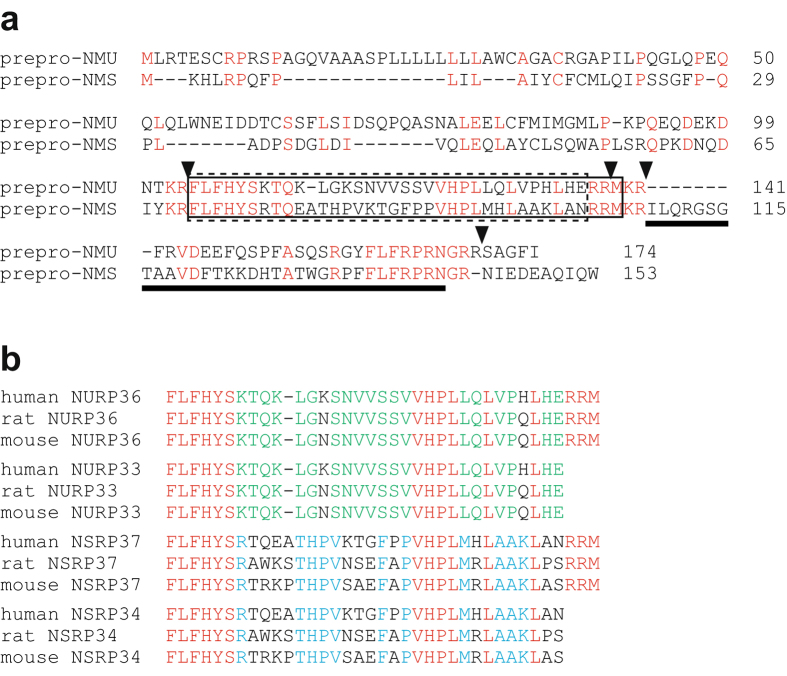
Structure of NURP. (**a**) Comparison of amino acid sequences between the human NMU precursor, prepro-NMU (accession number NM_006681), and the human NMS precursor, prepro-NMS (NM_001011717). Identical residues are shown in red. The arrowheads indicate proteolytic cleavage sites conserved in both precursors. The sequences corresponding to putative mature NURP33 and NSRP34 are surrounded by dotted lines, and those for mature NURP36 and NSRP37 are boxed by solid lines. The sequences of NMU and NMS are indicated by solid underlines. (**b**) Structures of mature NURP and NSRP peptides. Amino acid identities between NURP and NSRP are shown in red. The residues conserved only within the NURP or NSRP are indicated in green and blue, respectively.

Four potential proteolytic cleavage sites are conserved in the NMU precursors from mammals to fish (Supplementary Fig. S1) and in the mammalian NMS precursors (Fig. 1a)^14^. The comparison between precursor sequences of NMU and NMS showed that, in human, the amino acid sequences flanked by the first and second sites (numbered from the N-terminus of the precursor) exhibit considerably higher sequence identity to each other (42.4%) than the overall sequence identity (32.2%) (Fig. 1a). Moreover, these sequences had no homology to the sequences of previously known peptides or proteins. These data suggested the existence of novel biologically active neuropeptides within the precursors for NMU and NMS. We named these neuropeptides NURP and neuromedin S precursor-related peptide (NSRP), and we deduced their structures as follows. Because the second and third cleavage sites are very close together (Fig. 1a), C-terminal cleavage at these two sites would produce peptides that differed by only a few residues. The removal of the C-terminal basic residues by carboxypeptidase E^1^ would yield mature NURPs of 33 and 36 residues (designated NURP33 and NURP36, respectively) from the NMU precursor (Fig. 1b). Similarly, 34-residue and 37-residue NSRPs (designated NSRP34 and NSRP37, respectively) could be produced from the NMS precursor (Fig. 1b). The six N-terminal residues of NURP were identical to those of NSRP, and the peptides share additional sequence homology in their C-terminal regions; however, NURP and NSRP have no sequence homology to previously known peptides, including NMU and NMS. Therefore, they appear to form a new family.

### Purification and structure determination of endogenous NURP

**Figure 2:**
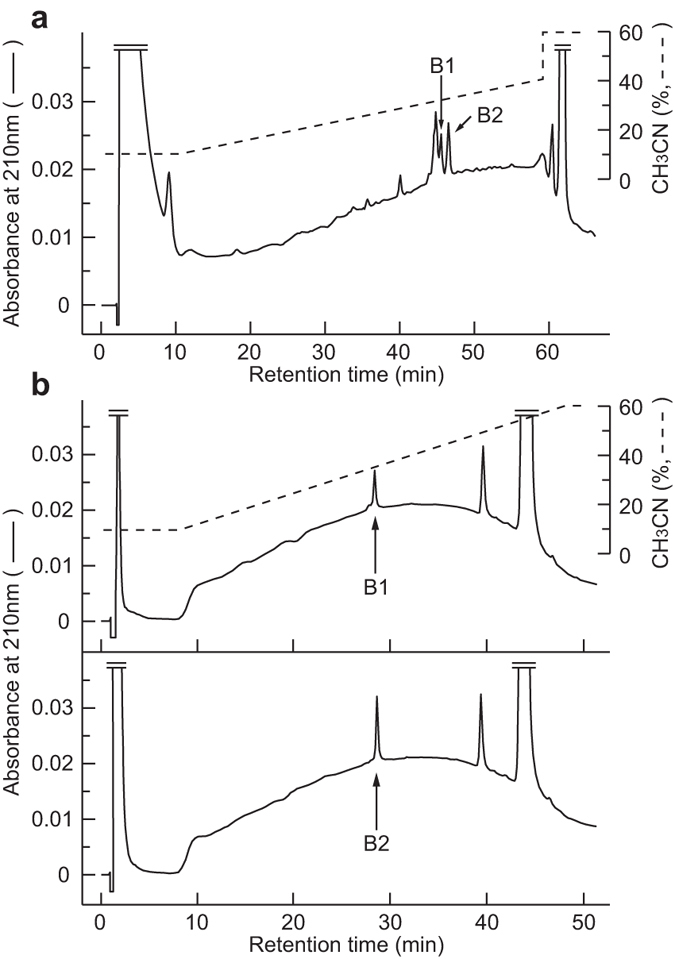
Purification of NURP from rat brain extracts. (**a**) RP-HPLC on a μ-Bondasphere C18 column of the materials eluted from the immunoaffinity column for rat NURP. The peaks containing NURP36 (B1) and NURP33 (B2) are indicated by arrows. (**b**) Final purification of rat NURP36 (upper; B1) and rat NURP33 (lower; B2) using a Chemcosorb 3ODS-H column. Purified peptides are indicated by arrows.

We purified endogenous NURPs from tissue extracts by using immunoaffinity chromatography followed by RP-HPLC. From extracts of 514 g of rat brain, two peptides (B1 and B2 in Fig. 2) were purified to homogeneity. The final yields of B1 and B2 were approximately 3.9 pmol and 7.3 pmol, respectively. The N-terminal sequences of the B1 and B2 peptides, FLFHYSKTQKLGNSNVVSSVVHPLLQLVPQL, were identical to each other and to the deduced N-terminal sequences of the NURPs (Fig. 1b). Mass spectrometric analysis revealed that the observed monoisotopic *m/z* values of B1 (4,202.2) and B2 (3,759.8) were very close to the theoretically predicted values for rat NURP36 (4,202.27) and rat NURP33 (3,759.03), respectively. These data indicate that B1 and B2 are natural peptides of rat NURP36 and NURP33, and that the deduced structures are identical to those of the natural peptides. In contrast, we were unable to purify endogenous NSRP from rat brain by using the same strategy as for NURPs.

Using the same methods as for rat brain, from 10 g of rat small intestine, we isolated three peptides: NURP36 (approx. 51.8 pmol, SI2), NURP33 (approx. 12.0 pmol, SI3), and oxidized NURP36 (approx. 71.2 pmol, SI1) (Supplementary Fig. S2). The structure of oxidized NURP36 was determiend by N-terminal sequencing (FLFHYSKTQKLGNSNVVSSVV) and mass spectroscopy (observed *m/z* value = 4,218.80).

### Immunochemical detection and quantification of NURP in rat tissues

To detect immunoreactive (ir)-NURP and ir-NMU in rat tissues, we developed RIAs for these peptides. According to the RIA standard curve for rat NURP, the half-maximum inhibition of tracer binding to antibody by rat NURP36 was 27 fmol/tube with the minimal limit of detection at 2 fmol/tube (Supplementary Fig. S3a). The antiserum used in this RIA (#24–6) was raised against a region common to NURP36 and NURP33, but it had no cross-reactivity with rat NSRP37 (Supplementary Fig. S3a), even though a six-residue sequence of the antigen peptide was identical to the N-terminal sequence of rat NSRP (Fig. 1b).

The RIA standard curve for rat NMU showed half-maximum inhibition at 4 fmol/tube, and the minimal limit of detection was 0.25 fmol/tube (Supplementary Fig. S3b). The antiserum used in the RIA for rat NMU (#14-4) had no cross-reactivity with rat NMS (Supplementary Fig. S3b), although the eight amidated C-terminal residues of the antigen peptide are identical to those of rat NMS^14^.

**Figure 3:**
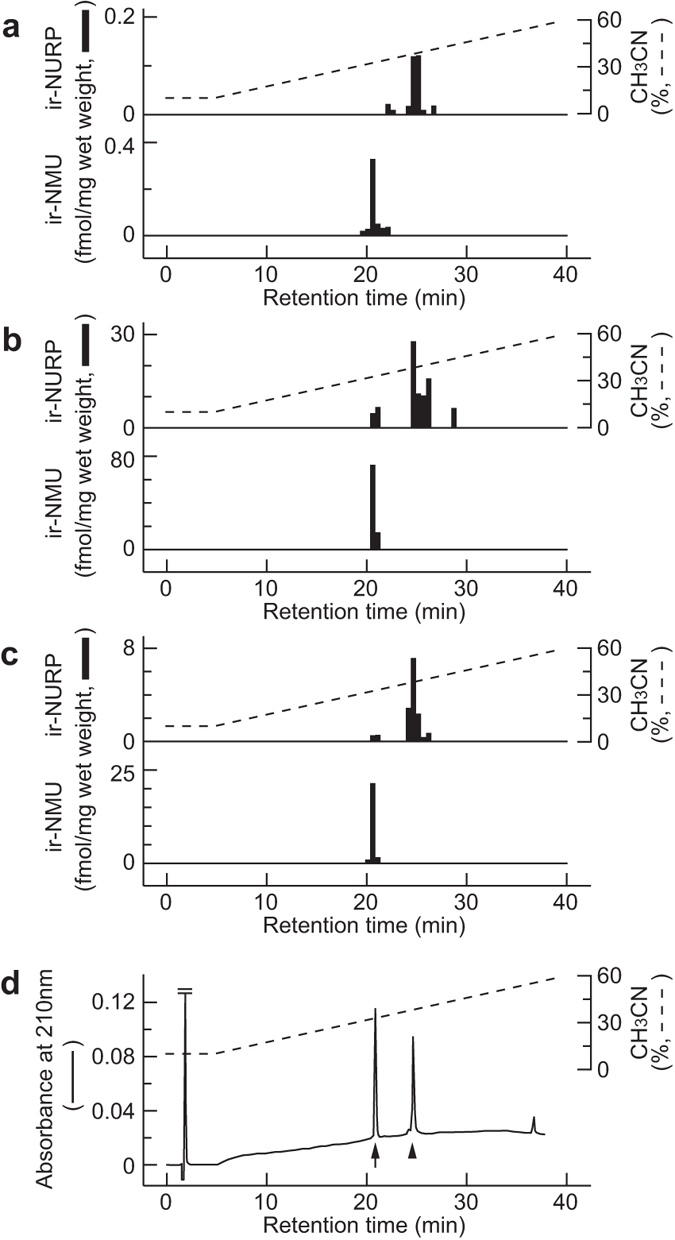
Representative RP-HPLC profiles of immunoreactivities for NURPs and NMU in rat tissues. (**a–d**) Peptide extracts from the whole brain (**a**), pituitary (**b**), and small intestine (**c**) of rat, and synthetic peptides (**d**) were applied to RP-HPLC under the same conditions. Samples of eluate were subjected to RIAs for rat NURP (upper, **a–c**) and for rat NMU (lower, **a–c**). Black bars indicate immunoreactivities (**a–c**). RP-HPLC profiles of immunoreactivities are each representative of three experiments. Elution positions of synthetic rat NURP36 and NMU are indicated by the arrowhead and arrow, respectively (**d**).

Because *NURP/NMU* mRNA apparently is expressed in the whole brain, pituitary, and small intestine of rats^8^, we determined the amounts of NURPs and the NURP:NMU ratios in these tissues. The molecular forms of ir-NURP and ir-NMU in the tissue extracts of rats were characterized by using RP-HPLC followed by RIA. In all tissue extracts tested, ir-NURP was detected as a broad peak with a retention time of 24.5 to 26 min (Fig. 3a–c). This broad peak probably contains several molecular species derived from NURP, because rat NURP36 was eluted within this retention time (25 min, Fig. 3d), whereas oxidized NURP36 was eluted slightly sooner; NURP33 was eluted slightly later than NURP36 from the same column under the same conditions except that an 80-min gradient was used (Supplementary Fig. S2). In contrast, ir-NMU was eluted as a sharp single peak, at a retention time (20.5 to 21 min) identical to that of synthetic rat NMU (Fig. 3a–d). The concentrations of ir-NURP and ir-NMU in these tissues were calculated from the results of three independent experiments (Table 1). Consistent with the expression pattern of *NURP/NMU* mRNA in rats^8^, both ir-NURP and ir-NMU were most abundant in the pituitary. Measurement of both immunoreactivities in the same samples indicated that the NURP:NMU ratios were similar among the various tissues tested (Table 1), with higher contents of NMU than total combined NURPs in each tissue.

**Table 1:** Immunoreactivities of NURPs and NMU in rat brain, pituitary, and small intestine.

Tissue	NURPs	NMU	NURPs/NMU
	(fmol/mg wet weight)		(ratio)
Whole brain	0.264 ± 0.009	0.394 ± 0.034	0.67
Pituitary	54.7 ± 7.26	70.0 ± 9.60	0.78
Small intestine	11.4 ± 0.75	22.3 ± 0.30	0.51

The amounts of total combined NURPs and of NMU in various tissues were quantified by RIA in combination with RP-HPLC fractionation. Data are presented as means ± SEM of three independent experiments.

In addition, we also developed RIA for rat NSRP (Supplementary Fig. S3c and Supplementary Methods). RIA coupled with RP-HPLC detected NSRP immunoreactivity in rat brain. The retention time at which this product eluted (20.5 to 21.5 min) was identical to that of synthetic rat NSRP37 (Supplementary Fig. S4). The concentration of ir-NSRP in rat brain was approximately 24 fmol/g wet weight.

### *In vivo* effects of NURP on prolactin release

**Figure 4:**
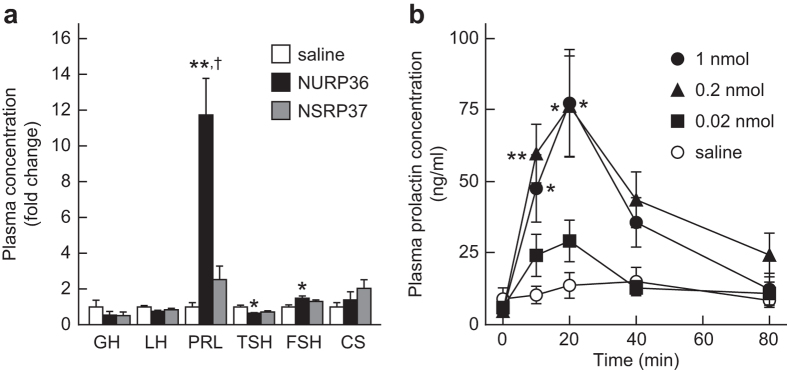
*In vivo* effects of NURP on prolactin release. (**a**) Effects of ICV-administered rat NURP36 on the plasma concentrations of anterior pituitary hormones and corticosterone in male rats. The plasma concentrations of the peptide-administered groups are shown as fold change relative to those of the saline-administered group. Data are presented as means ± SEM (*n* = 9 rats for the NURP36 and NSRP37 groups and *n* = 8 rats for the saline group). *P < 0.05; **P < 0.01 compared with the saline-administered group and ^†^ *P* < 0.01 compared with the NSRP37-administered group, one-way ANOVA followed by the Tukey–Kramer multiple-comparisons test. GH, growth hormone; LH, luteinizing hormone; PRL, prolactin; TSH, thyroid-stimulating hormone; FSH, follicle-stimulating hormone; CS, corticosterone. (**b**) Dose- and time-dependent effects of ICV-administered rat NURP36 on prolactin release in male rats. Rats were injected ICV with saline or NURP36 at the indicated doses. Data are presented as means ± SEM (*n* = 7 rats per group). *P < 0.05; **P < 0.01 compared with the saline-administered group at each time point, one-way ANOVA followed by the Tukey–Kramer multiple-comparisons test.

To understand the functional role of NURP, we validated its biological activity *in vivo*. Hypothalamic neuropeptides regulate the secretion of anterior pituitary hormones^18^, and *NURP/NMU* mRNA is highly expressed in the hypothalamo–pituitary region^9, 11^. Therefore, we first examined the effects of ICV administration of NURP on hormone release in conscious, male rats (Fig. 4a). The administration of 1 nmol NURP36 led to significant, robust increases in the plasma prolactin concentration (Fig. 4a; 78.96 ± 13.72 ng/ml [mean ± SEM] vs. 6.66 ± 1.76 ng/ml in the saline control). Although NURP36 induced weak but significant alterations in the plasma concentrations of thyroid-stimulating hormone and follicle-stimulating hormone, these significant differences were not reproducible. ICV-administered NURP36 had no significant effect on the plasma concentration of growth hormone, luteinizing hormone, or corticosterone, which is secreted from the adrenal cortex in response to adrenocorticotropic hormone secreted from the pituitary.

Furthermore, the increase in plasma prolactin concentrations induced by 1 nmol NURP36 in male rats also occured in female rats (26.15 ± 6.46 ng/ml [mean ± SEM; *n* = 4 rats] vs. 4.61 ± 3.24 ng/ml in the saline control [*n* = 4 rats], *P* < 0.05, Mann–Whitney test). As seen with NURP36, ICV-administered NURP33 induced significant increases in the plasma prolactin concentration of male rats (Supplementary Fig. S5). In contrast, ICV administration of 1 nmol rat NSRP37 to male rats did not significantly alter the plasma concentration of any of the hormones tested (Fig. 4a).

We then examined dose- and time-dependent effects in conscious, male rats (Fig. 4b). Saline administration failed to alter plasma prolactin concentrations. When rats were injected ICV with 0.02 nmol NURP36, prolactin release was stimulated slightly, but not significantly. Higher doses of NURP36 (0.2 and 1 nmol) induced significant, and similar, increases in the plasma prolactin concentrations (Fig. 4b), indicating that 0.2 nmol produces a maximal effect. Prolactin release peaked at 20 min and then gradually decreased. The prolactin-releasing activity of 0.2 nmol NURP36 was more potent than that of 0.2 nmol prolactin-releasing peptide (PrRP) (Supplementary Fig. S6).

### *In vitro* effect of NURP on prolactin release from anterior pituitary cells

**Figure 5:**
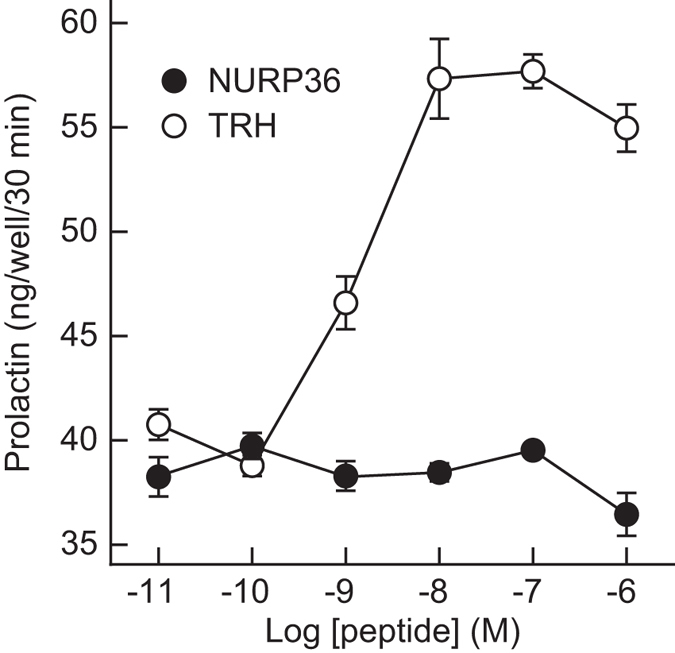
*In vitro* effect of NURP on prolactin release from anterior pituitary cells harvested from male rats. The cells were incubated for 30 min with increasing concentrations of rat NURP36 or TRH. Data are presented as means ± SEM (*n* = 3 wells per group). TRH, thyrotropin-releasing hormone.

To determine whether NURP36 acts directly on the lactotrophs of the pituitary, we next examined its effect on prolactin release from dispersed anterior pituitary cells harvested from male rats. NURP36 in log-molar concentrations ranging from 10 pM to 1 μM failed to alter prolactin release from anterior pituitary cells (Fig. 5). However, in parallel cultures of rat pituitary cells, prolactin release was stimulated in the expected concentration-dependent manner by the addition of thyrotropin-releasing hormone (Fig. 5), which acts directly on lactotrophs and induces prolactin release^18^.

### Effects of dopamine receptor agonist and antagonist on prolactin release induced by ICV-administered NURP

**Figure 6:**
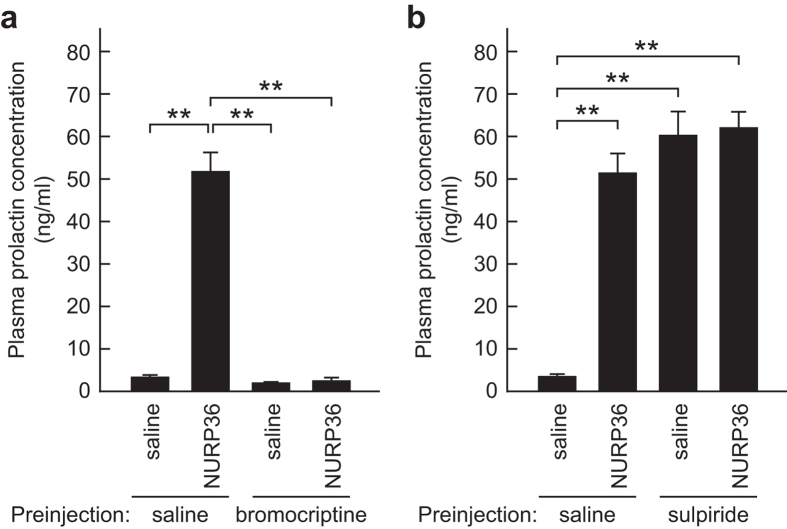
Effects of a dopamine receptor agonist and antagonist on prolactin release induced by ICV-administered NURP. (**a**) Effect of bromocriptine. Saline or rat NURP36 (1 nmol) was administered ICV to rats that had received a preinjection of saline or bromocriptine. Data are presented as means ± SEM (*n* = 5 rats per group). (**b**) Effect of sulpiride. Saline or rat NURP36 (1 nmol) was administered ICV to rats preinjected with saline or sulpiride. Data are presented as means ± SEM (*n* = 5 rats per group). **P < 0.01, one-way ANOVA followed by the Tukey–Kramer multiple-comparisons test. The same saline-pretreated groups were used in both panels.

Dopamine is a prominent inhibitor of prolactin release from the pituitary^18^. To clarify the relationship between the dopaminergic system and NURP, we examined the effects of a dopamine receptor agonist and an antagonist on prolactin release induced by ICV-administered NURP36. The prolactin-releasing activity of NURP36 was confirmed in male rats preinjected with saline (Fig. 6). However, treatment of male rats with bromocriptine, an agonist of the dopamine 2 receptor, followed by ICV administration of 1 nmol NURP36 did not increase plasma prolactin (Fig. 6a). Pretreatment of male rats with sulpiride, an antagonist of the dopamine 2 receptor, followed by ICV administration of 1 nmol NURP36 produced plasma prolactin concentrations similar to those in the saline-administered group (Fig. 6b). In this experiment, we used 1 nmol NURP36 instead of 0.2 nmol (Fig. 4b) to ensure that prolactin release was maximally and consistently stimulated in all rats.

### NURP does not activate NMURs

**Figure 7:**
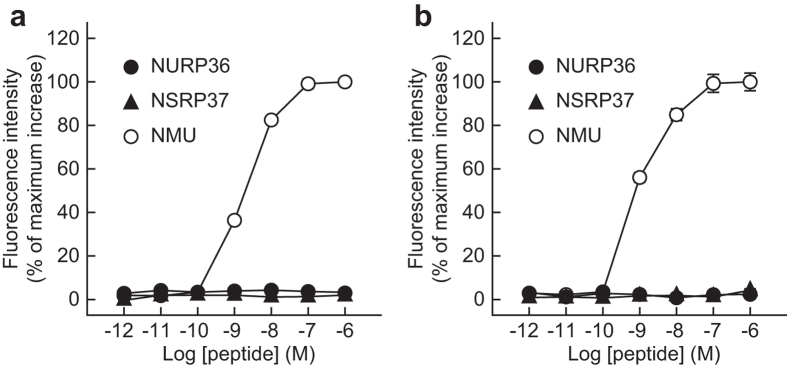
Pharmacological characterization of NURP by using CHO cells that stably express either rat NMUR1 or NMUR2. (**a**,**b**) Agonistic activities of rat NURP36, NSRP37, and NMU were evaluated by using the calcium-mobilization assay with CHO/rNMUR1-4 (**a**) and CHO/rNMUR2-11 (**b**) cells. Data points are presented as means ± SEM of triplicates for each experiment.

Because NURP is produced from the same precursor as NMU, we further examined whether NURP activates NMURs. Rat NMU induced dose-dependent, robust increases in the intracellular calcium ion concentration ([Ca^2+^]_i_) in CHO cells stably expressing either rat NMUR1 or NMUR2 (Fig. 7). In contrast, both rat NURP36 and NSRP37 failed to alter [Ca^2+^]_i_ in either cell line (Fig. 7). These data indicate that NURP and NSRP are unable to activate NMURs.

## Discussion

In this study, we biochemically identified NURP, a neuropeptide produced from the same precursor as NMU. NURP was detected immunochemically in the pituitary, small intestine, and brain—tissues that express *NMU* mRNA. Moreover, we showed that NURP is a hypothalamic prolactin-releasing neuropeptide that acts indirectly on the lactotrophs of the pituitary to induce prolactin release, possibly through the hypothalamic dopaminergic system.

After our discovery of NMU, the primary structures of the precursors for rat and human NMU were reported^19, 20^. Because consensus sequences for proteolytic cleavage additional to those involved in the generation of NMU were conserved in these precursors, the authors hypothesized that another peptide might be produced from the NMU precursor. However, biochemical evidence to support this hypothesis was unavailable at that time. Ten years later, we identified NMS, which is structurally related to NMU, and reported the conservation of four consensus sequences for cleavage between the precursors for NMU and NMS^14^. These data strongly suggested that the precursor for NMS might contain another peptide, as had been proposed for the precursor for NMU.

Although several secreted signalling peptides and proteins have been discovered by focusing on consensus sequences for precursor cleavage, the endogenous molecular forms have not been determined in some cases, such as for osteocrin (also known as musclin)^21, 22^. Because the endogenous molecular structure may influence the function, we were motivated to demonstrate that the neuropeptides we proposed (that is, NURP and NSRP) exist physiologically, and we here succeeded in the biochemical identification of endogenous NURP. We were unable to similarly purify endogenous NSRP from rat brain owing to its low content and the complicated oxidation reactions during the purification process. However, we were able to immunochemically identify NSRP in rat brain extract by using RP-HPLC followed by RIA, thus strongly suggesting that NSRP is present endogenously.

Understanding the regulatory mechanisms of anterior pituitary hormone secretion is one of the most important propositions in endocrinology, because these hormones play crucial roles in diverse physiological phenomena. Prolactin is predominantly synthesized and secreted from the lactotrophs of the pituitary^18^. Lactotrophs have high levels of spontaneous secretion, but the secretion is tonically inhibited, predominantly by dopaminergic neurons whose activity is governed by the hypothalamus^18^. However, increasing evidence indicates that unknown hypothalamic neuropeptides regulate prolactin release from the pituitary independently of—or in concert with—endogenous dopamine^23, 24^.

We propose that NURP is a candidate for such a hypothalamic neuropeptide. ICV-administered NURP36 specifically increased plasma prolactin concentrations in both male and female rats, and *NURP/NMU* mRNA is abundantly expressed in the rat hypothalamus^9, 11^. Therefore, NURP is a hypothalamic prolactin-releasing neuropeptide. However, NURP36 failed to stimulate prolactin release from dispersed anterior pituitary cells, indicating that it acts indirectly on lactotrophs to induce prolactin release. The prolactin-releasing activity of NURP36 was completely counteracted by pretreatment of rats with bromocriptine, a dopamine receptor agonist. Moreover, when the dopaminergic inhibition of prolactin release was blocked with sulpiride, ICV administration of NURP36 did not increase the plasma prolactin concentration more than administration of saline, suggesting that NURP does not affect prolactin release independently of the dopaminergic system. In contrast to dopamine, prolactin-releasing factors (PRFs), such as thyrotropin-releasing hormone, oxytocin, and vasoactive intestinal polypeptide, stimulate prolactin release by acting directly on lactotrophs^18^, and the serotonergic system in the brain promotes prolactin release by increasing PRF release^18^. Therefore, although it remains possible that PRFs mediate the prolactin-releasing activity of NURP, our data suggest that NURP exerts its activity through dopamine. Further investigation is needed to clarify the molecular mechanism by which the hypothalamic dopaminergic system might influence NURP-induced prolactin release.

Three populations of dopaminergic neurons in the hypothalamus are involved in the regulation of prolactin release from the pituitary^18^. One population is located in the periventricular nucleus, and the arcuate nucleus contains two distinct populations, the tuberohypophysial and tuberoinfundibular dopaminergic neurons. Because *NURP/NMU* mRNA is expressed in the arcuate nucleus^9, 11^, endogenous NURP might act on at least one of these dopaminergic populations to promote prolactin release. To establish the physiological significance of NURP in the regulation of prolactin release, the relationship between NURP-producing neurons and the dopaminergic system should be clarified through morphological analysis.

Previously, PrRP was identified as a potent, specific candidate for a PRF, because it promotes prolactin release from pituitary cells prepared from lactating female rats^25^. However, PrRP is unable to stimulate prolactin release from pituitary cells harvested from male rats^26^. In addition, ICV-administered PrRP fails to alter the plasma prolactin concentration in male rats, in which it does stimulate the secretion of luteinizing hormone and follicle-stimulating hormone^27^. Moreover, PrRP exerts a wide range of biological activities, including stress response and cardiovascular regulation^28^. Consistent with previous findings, plasma prolactin concentrations did not increase when we administered PrRP ICV to male rats. In contrast, ICV-administered NURP robustly induced prolactin release in both male and female rats. In addition, NURP is more potent than previously known peptidergic PRFs^18^ and induced maximal effect even at a low dosage (0.2 nmol).

Given that NURP and NSRP share regions of sequence homology, we were surprised that ICV-administered NSRP37 failed to induce the release of any of the anterior pituitary hormones we tested, including prolactin. Because endogenous NSRP was detected in rat brain, it will be interesting to clarify the central physiological role of this peptide. To this end, we are currently evaluating the biological activity of NSRP in the brain.

Unlike NURP, ICV-administered NMU decreases plasma prolactin concentrations^29^ and activates the dopaminergic neurons of the A12 cell group in the arcuate nucleus^30^. Therefore, a single precursor may produce two peptides, NURP and NMU, that have opposite actions on prolactin release, such that the ratio of NURPs to NMU may regulate prolactin release.

In tissues expressing *NURP/NMU* mRNA, both peptides were detected by RIA, and the NURP:NMU ratio was similar among the various tissues tested. Although both peptides are produced from the same precursor, the NURP content was lower than the amount of NMU in all tissues tested. The difference between the NURP and NMU contents can be explained by the following two mechanisms. First, the types and quantities of peptides produced from a precursor might depend on the kinds of subtilisin-like proprotein convertases that colocalize with the precursor during post-translational modification^1, 31^. Given its sequence, the first cleavage site, which is required for the production of NURP, is more suitable for PC1/3 and PC2 than for other members of subtilisin-like proprotein convertases. In contrast, the third and fourth sites, required for the production of NMU, can be cleaved by furin and furin-like proteases in addition to PC1/3 and PC2. Because fewer convertases are available to cleave the first site than the third and fourth sites, production of NURP may be lower than production of NMU, thus affecting the NURP:NMU ratio.

Another explanation for the differing amounts of NURP and NMU despite their common precursor involves alternative splicing of the primary *NMU* gene transcript. The coding sequence of the mammalian *NMU* gene is divided into ten exons, and NURP and NMU are encoded by exons 6–7 and 7–9, respectively^32^. Reverse transcription–polymerase chain reaction analysis and an EST database search revealed multiple splice variants of the *NMU* gene that lack exon 5 (30 bp), exon 6 (51 bp), or both exons, such as the cDNA clones with accession numbers BF563287, BF035464, and CN343322. These variants encode precursors can produce only NMU, because exons 5 and 6 encode the consensus sequence for the first cleavage site and the N-terminal sequence of NURP, respectively. Moreover, when either exon 8 (54 bp) or exon 9 (40 bp) is deleted (or both exons), the variants encode precursors specific for the production of NURP. Because the structure of the *NMU* gene is conserved among mammalian species, alternative splicing seems to be an important mechanism for determining the NURP:NMU ratio. In support of this notion, the production of neuropeptides derived from preprotachykinin is regulated by alternative splicing^33^. Because the NURP:NMU ratio *in vivo* may be important for regulating the release of prolactin from the pituitary, clarifying the molecular mechanism that controls this ratio will be important for understanding how these peptides modulate prolactin release.

NURP and NMU were most abundant in rat pituitary. Although NMU acts directly on pituitary cells to suppress gonadotropin release, thus regulating the onset of puberty^34^, NURP was unable to act directly on lactotrophs to induce prolactin release. Although NURP might have a direct effect on the pituitary, this role—like the physiological significance of NURP in the pituitary—remains elusive.

Our previous work^14^ implied the existence of the novel neuropeptides that we here designated NURP and NSRP. A previous study^35^ demonstrated the biological activity of NURP prior to confirmation that this peptide is present endogenously. Specifically, ICV administration of the synthetic peptide corresponding to NURP induced a transient increase in feeding in mice, after which food intake then decreased over the following 24 h. Together with our data, this previous report^35^ strongly supports that NURP has biological activity.

Identification of the receptor for NURP will be critical for clarifying its physiological role, because in many cases the physiological significance of signalling molecules for which receptors have not yet been identified is poorly understood. The receptors for neuropeptides and peptide hormones are primarily GPCRs. Because NURP is produced from same precursor as NMU, we first examined whether NMURs, which belong to class A of the GPCR superfamily^13^, function as receptors for NURP. However, NURP did not activate these receptors in the cell-based assay we used. We are currently investigating whether NURP activates various orphan GPCRs but have not yet identified the receptor for NURP.

Our identification and characterization of NURP, together with the biological evidence that it is present endogenously, provide a foundation for further research into the mechanism by which prolactin is released from the pituitary.

## Methods

### Synthetic peptides

Rat NURP36, rat NURP33, rat NSRP37, [Cys^0^]-rat NURP [1–20], [Cys^0^]-rat NSRP [1–20], and rat NMU were chemically synthesized by using a model 431A peptide synthesizer (Applied Biosystems). [Tyr^21^]-rat NURP [1–20] was synthesized by Invitrogen. [Tyr^35^]-rat NSRP34 was synthesized by Sigma. The peptides synthesized were purified to a single peak by RP-HPLC. The structures of these synthetic peptides were confirmed by sequencing analysis and mass spectrometry. The amounts of peptides were determined by amino acid analysis. Thyrotropin-releasing hormone and rat PrRP were purchased from Peptide Institute, Inc.

### Animals

All procedures were performed in accordance with the Japanese Physiological Society’s guidelines for animal care, and the experiments were approved by the Animal Care and Use Committee of the National Cerebral and Cardiovascular Center Research Institute (authorization number 16040) and the Animal Experiment Committee of the University of Miyazaki (authorization number 2012-006-5). Animals used in this study were obtained from SLC Japan Inc. and CLEA Japan Inc. and maintained in individual cages under controlled conditions of temperature (22 ± 1 °C) and lighting (lights on, 07:00 to 19:00) with unrestricted access to chow and water.

### Preparation of antibodies

The antisera used in this study were obtained as described previously^36^ with minor modifications. The antiserum for rat NURP was raised against the N-terminal fragment (positions 1–20) of NURP, the antiserum for rat NMU was raised against the whole molecule of rat NMU, and the antiserum for rat NSRP was raised against the N-terminal fragment (positions 1–20) of NSRP. [Cys^0^]-rat NURP [1–20] and [Cys^0^]-rat NSRP [1–20] were respectively conjugated to maleimide-activated mariculture keyhole limpet hemocyanin (KLH; Pierce). Rat NMU was conjugated to mariculture KLH by carbodiimide chemistry. New Zealand white rabbits were immunized separately with each of these conjugates emulsified in adjuvant.

### Preparation of an immunoaffinity column for the purification of endogenous NURP

The immunoglobulin G specific for NURP was purified from antiserum #24-6 using a Protein A Sepharose column (GE Healthcare) and then immobilized to Affi-Gel 10 (BioRad) at a concentration of 6.5 mg/ml gel in accordance with the manufacturer’s instructions. The binding capacity of this immunoaffinity column is 0.98 nmol rat NURP36/ml gel.

### Purification and structure determination of endogenous NURP

The peptide fractions (SP-III) were extracted from fresh rat brain (514 g) and small intestine (10 g) as described previously^14^. The lyophilized SP-III fractions were dissolved in 0.1 M sodium phosphate buffer (pH 7.4) and then loaded onto an immunoaffinity column (gel volume, 3 ml) equilibrated with the same buffer. After the column was washed, the absorbed materials were eluted with 1 M acetic acid containing 10% CH_3_CN and re-purified on the same column. The eluted materials from the brain were separated by RP-HPLC on a μ-Bondasphere C18 column (2.1 × 150 mm; Waters) at a flow rate of 0.2 ml/min with a gradient of CH_3_CN in 0.1% trifluoroacetic acid (TFA) as indicated in Fig. 2a. The materials from the small intestine were also separated on a Symmetry 300 C18 column (3.9 × 150 mm; Waters) with a linear gradient from 10% to 60% of CH_3_CN in 0.1% TFA for 80 min at a flow rate of 1 ml/min. Finally, each peak was purified to homogeneity by RP-HPLC on a Chemcosorb 3ODS-H column (2.1 × 75 mm; Chemco) with a linear gradient from 10% to 60% of CH_3_CN in 0.1% TFA for 40 min at a flow rate of 0.2 ml/min. The column eluates were monitored continuously by measuring the absorbance at 210 nm, and the yield of purified peptide was determined from the absolute absorbance. The primary structure of the purified peptide was determined by using a protein sequencer (model 494 cLC, Applied Biosystems). The correct mass of the purified peptide was verified by matrix-assisted laser desorption–ionization time-of-flight mass spectrometry on a Voyager-DE PRO mass spectrometer (Applied Biosystems).

### Immunochemical detection and quantification of NURP and NMU in rat tissues

The contents of NURP and NMU in rat tissues were measured by performing RIA coupled with RP-HPLC as described previously^37^. In brief, tissue extracts were prepared from whole brain, pituitary, and small intestine of male Wistar rats (10 weeks old). Samples equivalent to 1,200 mg wet weight of whole brain, 0.6 mg wet weight of pituitary, and 24 mg wet weight of small intestine were separated by RP-HPLC on a Symmetry 300 C18 column (3.9 × 150 mm; Waters) with a linear gradient from 10% to 60% of CH_3_CN in 0.1% TFA for 40 min at a flow rate of 1 ml/min. The eluates were collected every 30 sec. To determine the peptide contents in the eluate, eluate samples equivalent to 500 mg wet weight of whole brain, 0.25 mg wet weight of pituitary, and 10 mg wet weight of small intestine were subjected to RIA for rat NURP. In addition, portions of the same eluate equivalent to 50 mg wet weight of whole brain, 0.025 mg wet weight of pituitary, and 1 mg wet weight of small intestine were subjected to RIA for rat NMU. For RIA, [^125^I-Tyr^21^]-rat NURP [1–20] and radioiodinated rat NMU were used as tracer ligands, and antisera for rat NURP (#24-6) and NMU (#14-4) were used at final concentrations of 1/12,000 and 1/380,000, respectively. Known amounts of rat NURP36 and rat NMU were used to obtain standard curves.

### *In vivo* experiments

A 23-gauge stainless-steel guide cannula was implanted into one of the lateral ventricles of anesthetized Wistar rats (8–9 weeks old), as described previously^38^. The cannula-implanted rats were housed in individual cages for 1 week, after which a microinjection cannula was used to inject 10 μl of saline with or without peptide through the implanted guide cannula into conscious rats between 09:00 and 09:30. To examine the effects of ICV administration of peptides or saline (vehicle), blood samples were collected by rapid decapitation 20 min after administration. In the time-course experiments, the blood samples were collected by tail-tip incision before and at 10, 20, 40, and 80 min after injection. In the experiments involving the dopamine receptor agonist and antagonist, bromocriptine (1 mg/kg body weight), sulpiride (1 mg/kg body weight), or saline was injected intraperitoneally 10 min before peptide administration, and blood samples were collected by tail-tip incision 20 min after injection of NURP36 or saline.

### *In vitro* experiment

Dispersed anterior pituitary cells were prepared from male Wistar rats (5 weeks old) in accordance with a previously described method^39^. The cells were plated at 5 × 10^4^ cells/well in poly-L-lysine–coated 96-well tissue-culture plates containing DMEM supplemented with 10% fetal calf serum and a mixture of antibiotics and cultured at 37 °C in an atmosphere of 5% CO_2_. After 2 days, the conditioned medium was removed and replaced with test medium (DMEM containing 0.1% bovine serum albumin and 2.5 mM bacitracin) only or containing peptide sample. After incubation at 37 °C for 30 min, the test medium was collected for subsequent determination of prolactin content.

### RIA for hormones

To measure the concentration of hormones, plasma was collected from blood samples by centrifugation. The conditioned medium of anterior pituitary cells was diluted with phosphate-buffered saline to a suitable concentration. The concentration of hormones in the plasma and conditioned medium was measured with Biotrak assay systems (GE Healthcare) for rat prolactin, luteinizing hormone, follicle-stimulating hormone, growth hormone, thyroid-stimulating hormone, and corticosterone. Because Biotrak assay systems became commercially unavailable, the concentration of prolactin was measured by using rat prolactin enzyme immunoassay kit (Bertin Pharma).

### Cell-based assay

CHO cells stably expressing rat NMURs were established as described in the Supplementary Information. The calcium-mobilization assay was performed by using the FLIPR system (Molecular Devices) as described previously^14^.

### Statistical analysis

Data are presented as means ± SEM. Differences between groups were analysed by using the nonparametric Mann–Whitney test. Statistical comparisons between more than two groups were examined by one-way analysis of variance (ANOVA) followed by the Tukey–Kramer multiple-comparisons test. *P* < 0.05 was considered statistically significant.

### Data availability

All sequences of NURPs and NSRPs have been submitted as third party data to the DDBJ/EMBL/GenBank databases and assigned accession numbers BR001408-BR001413. The amino acid sequences of rat NURP36 and rat NURP33 have also been submitted to the UniProt Knowledgebase under accession number P12760.

## Electronic supplementary material

Supplementary Information
