# Supplementary material for: Identification of neuromedin U precursor-related peptide and its possible role in the regulation of prolactin release
Source: Sci Rep. 2017 Sep 5;7:10468. doi: 10.1038/s41598-017-10319-9 (PMC5585327; doi:10.1038/s41598-017-10319-9)
Supplement: Supplementary file 1 — Supplementary Information [file 41598_2017_10319_MOESM1_ESM.pdf]

Supplementary Information for

Identification of neuromedin U precursor-related peptide and its possible role in the regulation of prolactin release

Kenji Mori, Takanori Ida, Mami Fudetani, Miwa Mori, Hiroyuki Kaiya, Jun Hino, Keiko Nakahara, Noboru Murakami, Mikiya Miyazato, Kenji Kangawa

Correspondence should be addressed to Kenji Kangawa (kangawa@ri.ncvc.go.jp)

Supplementary Methods

Establishment of CHO cells expressing either rat NMUR1 or NMUR2

Full-length cDNAs of rat NMUR1 and NMUR2 were amplified by reverse transcription–polymerase chain reaction by using total RNA prepared from rat brain. The primer set used for rat NMUR1 was 5'-TTCCCCTGCAGAAGGCATGCTCTC-3' and 5'-AGGGCCTAGCGCTGAAGTTACAGG-3', and for rat NMUR2 was 5'-AGCTCAGTAATGGGAAAACTTG-3' and 5'- ATGGATGTCAATCACAGAATCC-3'. Amplified cDNAs were cloned into pcDNA3.1 vector (Invitrogen), and then the sequences of cDNAs were confirmed. CHO cells were transfected with the expression plasmid, after which they were maintained in a culture medium containing 1 mg/ml G418 to select for stable transfectants. After 2 weeks, 12 colonies containing each plasmid were picked, and the expression of receptor mRNA was validated by quantitative polymerase chain reaction analysis. Stably expressing cell lines (CHO/rNMUR1-4 and CHO/rNMUR2-11) were obtained, and the function of the recombinant receptors expressed in these cell lines was confirmed by the calcium-mobilization assay with rat NMU.

Immunochemical detection of NSRP in rat brain

A tissue extract was prepared from whole brain of male Wistar rats (10 weeks old). A sample of this extract equivalent to 9.1 g wet weight of whole brain was separated by gel filtration HPLC on a TSKgel G2000SWxl column (21.5 × 300 mm; TOSOH) equilibrated with 23% CH_3CN containing 0.1% TFA at a flow rate of 2 ml/min. The eluate was collected between 32 and 46 min. This fraction contains peptides that have molecular

weights between approximately 1,000 and 14,000 Da. The collected fraction was concentrated by using a Sep-Pak C18 cartridge (Waters), and then separated by RP-HPLC on a Symmetry 300 C18 column (3.9 × 150 mm; Waters) with a linear gradient from 10% to 60% of CH_3CN in 0.1% TFA for 40 min at a flow rate of 1 ml/min. Samples of eluate were collected every 30 sec. To determine the peptide contents in the eluate, samples equivalent to 1 g wet weight of whole brain were subjected to RIA for rat NSRP. For RIA, [^{125}I-Tyr^{35}]-rat NSRP34 was used as the tracer ligand, and antiserum for rat NSRP (#29-6) was used at final concentration of 1/24,300. Known amounts of rat NSRP37 were used to obtain the standard curves.

On the standard RIA curve for rat NSRP, the half-maximum inhibition of tracer binding to antibody by rat NSRP37 was 23 fmol with the minimal limit of detection at 4 fmol (Supplementary Fig. S3c). The antiserum used in this RIA (#29-6) had no cross-reactivity with rat NURP36 (Supplementary Fig. S3c), even though a six-residue sequence of the antigen peptide was identical to the N-terminal sequence of rat NURP (Fig. 1b).

Supplementary Fig. S1

```
human           MLR---TE--SCRPRSPAGQVAA--ASPLLLLLLLLAWCAGACRGAPILPQGLQPEQQLQLWNEIDDTCS 63
marmoset        M------------------------------------------AGAPILPQGLQPDQQLQLWNEIDDACS 28
cow             MLR---AA--SRRPEPPAGHVAA--GSPLLLL-LLLSCCADACGGAPVLPQGLQPEQELQLWKQINDACL 62
mouse           MSR---AA--GHRPGLSAGQLAAATASPLLSLLLLLACCADACKGVPISPQRLQPEQELQLWNEIHEACA 65
rat             MSR---AA--NRRPGLSAGQLAAATASPLLSLLLLLACCADACRGTPISPQRLPPEQELQLWNEIPEACA 65
chicken         MARLCRQQLHAAIPPRGPGSGWGLPGSPLLLLLFLLASSVRVCKGVPMPSQALEAEQELQLWKEIDDACS 70
tree frog       MQKGSEDT---TQNRCHQHSIGGHSTCG-LLLLIILVSWTSICEGAPFSSPVLGAEDELPLWNGIDDACS 66
goldfish        MRSSS-----QCERGAAQRAMSPAHNSA-LMLGVLLISCIPITTSAPVLLNPSSIE-HEQLLTQITDLCS 63

                                                        ▼
human           SFLSIDSQPQASNALEELCFMIMGMLPKPQEQDEKDNTKRFLFHYSKTQKLGKSNVVSSVVHPLLQLVPH 133
marmoset        SFLSVDSQPQASNALEELCFVIMGMLPKPQEQDEKDNTKRFLFHYSKTQKLGKSNVVSSVVHPLLQLVPR 98
cow             SLLSMHPQPQASNALEEICLTIMRTLPKPQETDEKDNTKRFLFHYSKTQKLGNSNVVSSVLHPLLQLVPQ 132
mouse           SFLSIDSRPQASVALRELCRIVMEISQKPQEQSEKDNTKRFLFHYSKTQKLGNSNVVSSVVHPLLQLVPQ 135
rat             SFLSVDSQPQASVALRKLCRVLMEIFQKPQEQTEKDNAKRFLFHYSKTQKLGNSNVVSSVVHPLLQLVPQ 135
chicken         AYMSTDSQPQVSSTLEELCFLAMGFLQKPQGSDEKDNMKRFLFHYSKTHDSGNSDVRSSVLHPLLQLVPQ 140
tree frog       AVLP-DPQLAVSSTLRELCFMVMRMQQKSQG-EEKDDFKRFLFHYSKSHDSGNSDITSSVLHPLLQLLPQ 134
goldfish        FYLSADPSFRTSDVLEDLCFLMLGSLQKSKEITARETRKRFLFHYTKPNGAGLSDGTSTVLHPLLELIPH 133

                     ▼  ▼                           ▼
human           LHERRMKRFRVDEEFQSPFASQSRGYFLFRPRNGRRSAGFI 174
marmoset        LHERRMKRFGLDEEFQSPFASQSRGYFLFRPRNGRRSTGFI 139
cow             LHERRMKRFRLDEEFQGPIASQSRRYFLFRPRNGRRSEGYI 173
mouse           LHERRMKRFKA-E-YQSPSVGQSKGYFLFRPRNGKRSTSFI 174
rat             LHERRMKRYKVNE-YQGP-VAPSGGFFLFRPRNGKRSTSFI 174
chicken         LNERRLKRYKVDEDLQGAGGIQSRGYFFFRPRNGKRSVDFR 181
tree frog       LHDRRMKRLTSDEEVQVPGGVISNGYFLFRPRNGRRSAGFR 175
goldfish        LARRRSRRMKLNDDLQGPGRIQSRGFFLYRPRNGRRSDEYV 174
```

Supplementary Figure S1. Amino acid sequences of vertebrate prepro-NMU. The typical consensus sequences for precursor cleavage (Arg–Arg and Lys–Arg) are shown in red, and proteolytic cleavage sites are indicated by arrowheads. Accession numbers: human, NM_006681; marmoset, AY057098; cow, NM_001191153; mouse, NM_019515; rat, NM_022239; chicken, NM_001277921; tree frog, AJ457825; goldfish, AB499530.

Supplementary Fig. S2

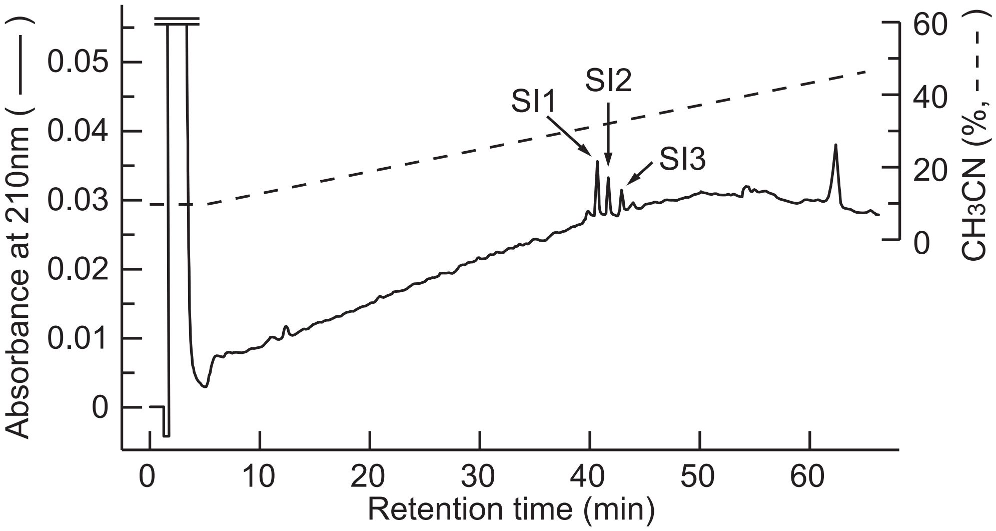

Supplementary Figure S2. Purification of NURP from rat small-intestine extracts. The materials eluted from the immunoaffinity column for rat NURP were separated by RP-HPLC on a Symmetry 300 C18 column. The peaks containing oxidized NURP36 (SI1), NURP36 (SI2), and NURP33 (SI3) are indicated by arrows.

Supplementary Fig. S3

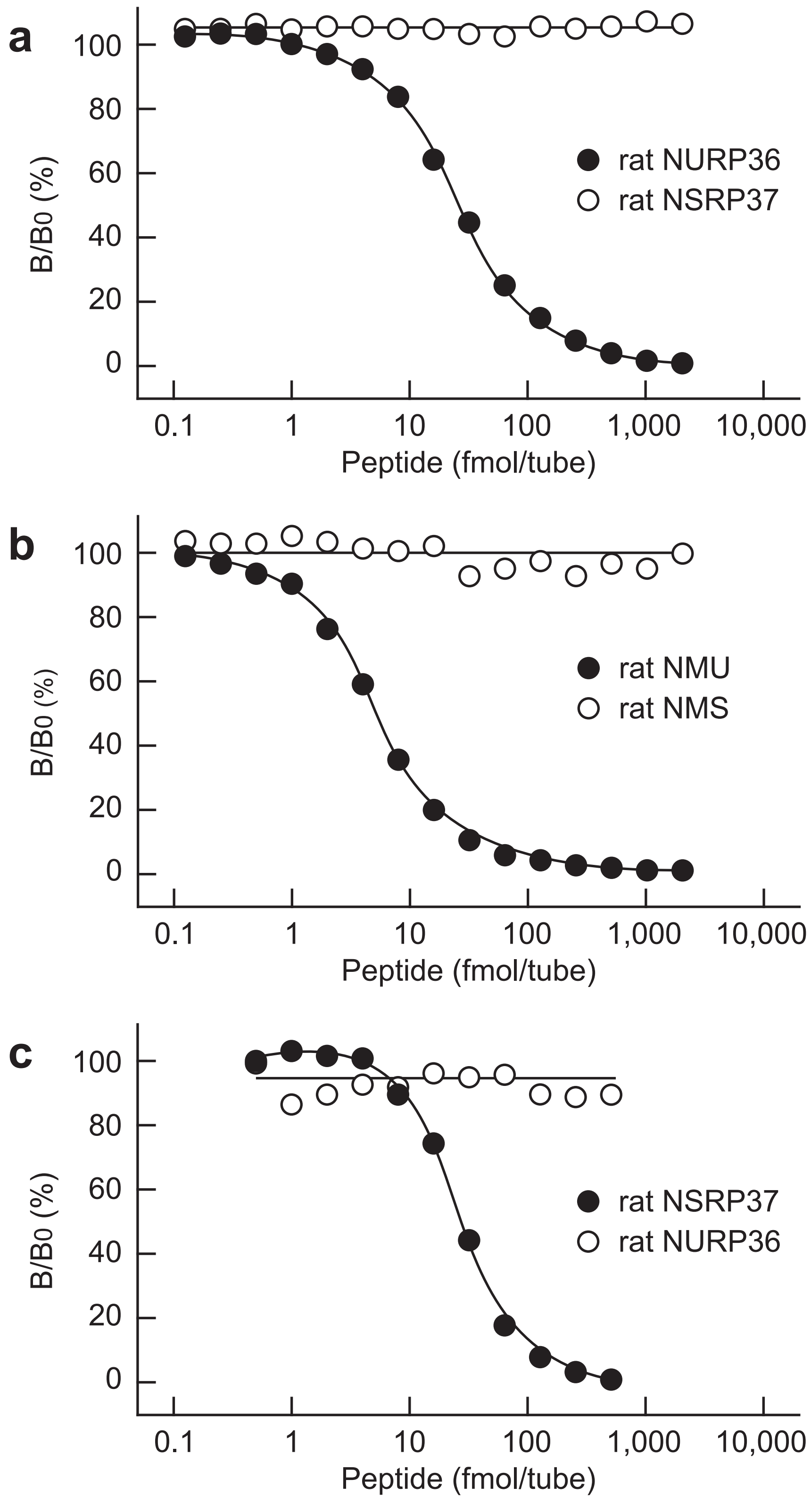

Supplementary Figure S3. Representative standard curves from the RIAs of rat NURP, NMU, and NSRP. (**a**) [^{125}I-Tyr^{21}]-rat NURP [1–20] binding to antibody #24-6 was displaced by increasing concentrations of rat NURP36 but not rat NSRP37. (**b**) Radioiodinated rat NMU bound to antibody #14-4 was displaced by increasing concentrations of rat NMU but not rat NMS. (**c**) [^{125}I-Tyr^{35}]-rat NSRP34 binding to antibody #29-6 was displaced by increasing concentrations of rat NSRP37 but not rat NURP36. B/B0 means tracer bound/tracer bound in zero standards. The amounts of peptides (X-axis) are expressed on a log scale. These standard curves for rat NURP36, NMU, and NSRP37 are each representative of four experiments.

Supplementary Fig. S4

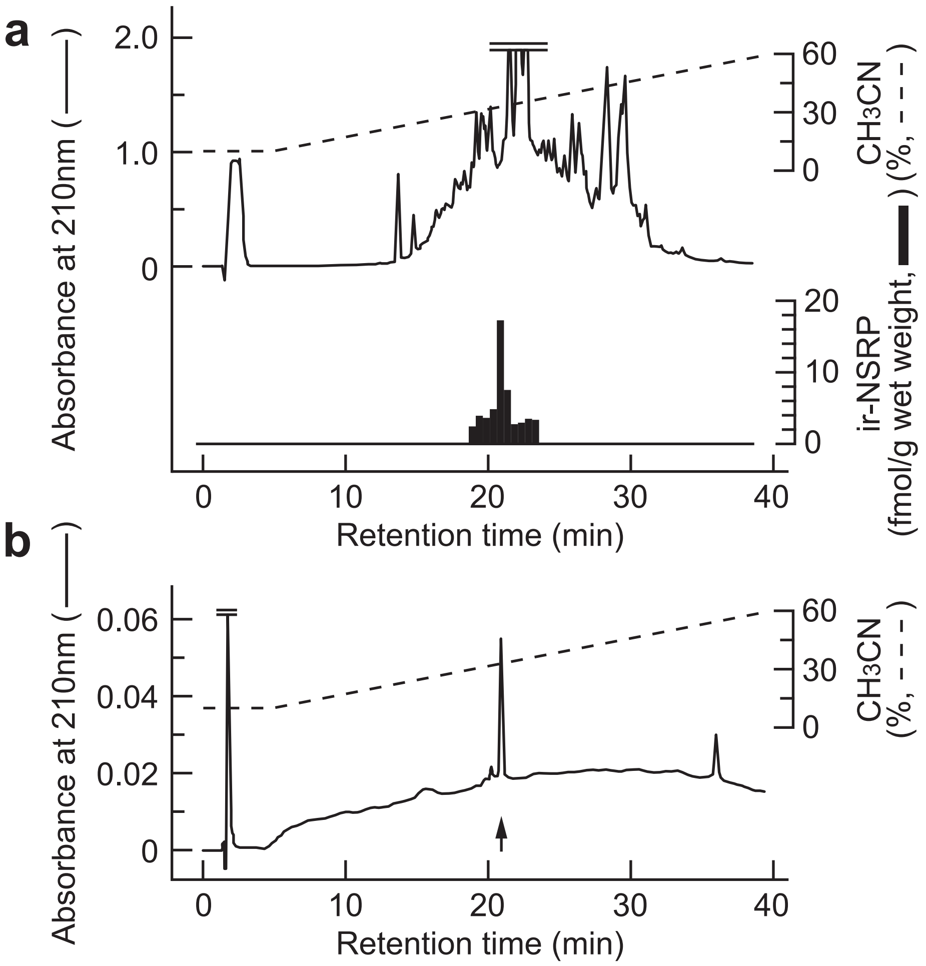

Supplementary Figure S4. Representative RP-HPLC profile of immunoreactivity for NSRP in rat brain. (**a**) After gel-filtration HPLC, peptide extract from rat brain was separated by RP-HPLC. Samples of eluate were subjected to RIA for rat NSRP. Black bars indicate immunoreactivities. The RP-HPLC profile of immunoreactivities is representative of two experiments. (**b**) Synthetic rat NSRP37 was separated under the same conditions. The elution position of synthetic rat NSRP37 is indicated by the arrow.

Supplementary Fig. S5

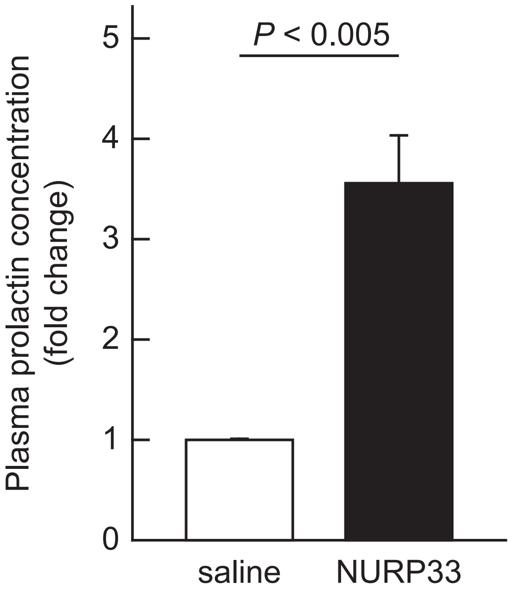

Supplementary Figure S5. *In vivo* effect of NURP33 on prolactin release in male rats. Rats were injected ICV with saline or 1 nmol rat NURP33. The plasma concentration of the NURP33-administered group is shown as fold change relative to that of the saline-administered group. Data are presented as means ± SEM (n = 6 rats per group, Mann–Whitney test).

Supplementary Fig. S6

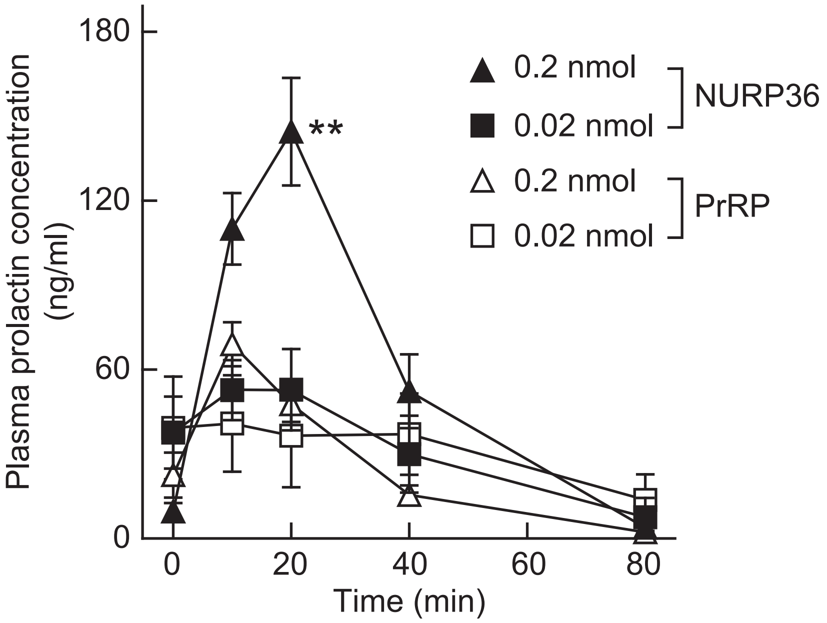

Supplementary Figure S6. Dose- and time-dependent effects of ICV-administered rat NURP36 and prolactin-releasing peptide (PrRP) on prolactin release in male rats. Rats were injected ICV with rat NURP36 at the dose of 0.2 nmol (n = 3 rats) or 0.02 nmol (n = 3 rats), or with PrRP at the dose of 0.2 nmol (n = 4 rats) or 0.02 nmol (n = 5 rats). Data are presented as means ± SEM. **, $P < 0.01$ compared with the same dose of the PrRP-administered group at the same time point (one-way ANOVA followed by the Tukey–Kramer multiple-comparisons test).
